# Exercise- and diet-induced glycogen depletion impairs performance during one-legged constant-load, high-intensity exercise in humans

**DOI:** 10.3389/fphys.2025.1564523

**Published:** 2025-08-15

**Authors:** Martin Thomassen, Michael J. McKenna, Hugo Olmedillas, Victoria Wyckelsma, Jens Bangsbo, Nikolai Baastrup Nordsborg

**Affiliations:** ^1^ The August Krogh Section for Human Physiology, Department of Nutrition, Exercise and Sports, University of Copenhagen, Copenhagen, Denmark; ^2^ Institute for Health and Sport, Victoria University, Melbourne, VIC, Australia; ^3^ College of Physical Education, Southwest University, Chongqing, China; ^4^ College of Sport Science, Zhuhai College of Science and Technology, Zhuhai, China; ^5^ Department of Functional Biology, University of Oviedo, Oviedo, Spain; ^6^ Department of Physiology and Pharmacology, Karolinska Institute, Stockholm, Sweden

**Keywords:** MVC, potassium, Na^+^/K^+^-ATPase, FXYD1, excitability

## Abstract

**Introduction:**

The effect of muscle glycogen stores on performance during intense short-duration exercises in humans is unclear. We hypothesized that low initial muscle glycogen levels would impair constant-load intense one-legged knee extensor exercise lasting approximately 5 min and human muscle contractile function, as determined by maximal voluntary contraction (MVC), electrically induced single-twitch maximal force, rate of force development (RFD), and rate of relaxation. Furthermore, alter phosphorylation of the Na^+^/K^+^-ATPase (NKA) regulatory proteins AMPK and FXYD1 indicating attenuated NKA activity.

**Methods:**

With one leg in a glycogen-depleted state and one leg in a glycogen-loaded state, ten healthy young males (age: 25 ± 2 years) performed three intense exercise trials including (i) two-legged cycling for ∼5 min and (ii) 2× one-legged knee extensor exercise to task failure. MVC determination, electrical muscle stimulation, blood sample testing, and vastus lateralis biopsies were performed to assess the muscle composition and function.

**Results:**

Time to task failure during the one-legged knee-extensor exercise was reduced by approximately 40% (n = 10, *P* < 0.05) with exercise- and diet-induced glycogen depletion. At rest (n = 10), MVC, twitch force, RFD, and rate of relaxation were unaffected by glycogen content. After exercise to task failure, the single-twitch contractile characteristics were impaired to a greater extent (n = 10, *P* < 0.05) in the glycogen-loaded leg than in the glycogen-depleted leg, probably induced by longer exercise duration. Concomitantly, MVC (n = 10, except for 15 s: n = 5 and 8) was reduced to similar levels under both conditions. The exercise-induced increase in the nonspecific phosphorylation of FXYD1 (n = 10, *P* < 0.001), which regulates NKA activity, tended to be greater (n = 10, *P* = 0.06) in the glycogen-loaded legs, indicating augmented potassium handling.

**Conclusion:**

Exercise- and diet-induced low muscle glycogen content impairs high-intensity constant-load exercise performance lasting approximately 5 min. This was observed even with concomitantly better single-twitch contractile characteristics and similar reduction in MVC after task failure compared to glycogen-loaded legs. At rest, glycogen levels did not affect MVC and contractile characteristics.

## 1 Introduction

Task failure is related to reaching critically low muscle glycogen levels during exercise at a broad range of intensities (Bergstrom et al., 1967; Coyle et al., 1986; Greenhaff et al., 1987; Rockwell et al., 2003). At rest, approximately 400 mmol·kg_d.w._^−1^ of glycogen is stored in the human vastus lateralis muscle, and the absolute content is highly dependent on dietary interventions, training status, and preceding exercise. In highly trained endurance athletes, exhaustion due to very low muscle glycogen content can be postponed if carbohydrates are ingested during prolonged strenuous exercise (Coyle et al., 1986).

However, the importance of the glycogen content in relation to short-duration intense exercise with combined aerobic and anaerobic energy contribution remains unclear. A low initial glycogen content (<200 mmol·kg_d.w._^−1^) has been associated with impaired repeated 6 s maximal sprint performance (Balsom et al., 1999), whereas increasing the muscle glycogen content to approximately 700 mmol·kg_d.w_^−1^ has no effect on intense exercise performance lasting approximately 3 min (Bangsbo et al., 1992). Currently, the importance of an initially low muscle glycogen content for intense constant-load exercise capability is unclear (Vigh-Larsen et al., 2022), and no studies have included direct measurements of the muscle glycogen content. Therefore, the first aim of this study was to examine the impact of exercise- and diet-induced low muscle glycogen content on performance during brief (∼5 min) constant-load intense exercise with a pronounced aerobic and anaerobic energy production and a notable glycogen breakdown (Chidnok et al., 2013). We hypothesized that low initial muscle glycogen levels would impair performance determined as time to task failure in constant-load intense one-legged knee extensor exercise lasting approximately 5 min.

The mechanism by which low glycogen content compromises muscle function has not been fully elucidated (Vigh-Larsen et al., 2021). However, a reduced sarcoplasmic reticulum (SR) Ca^2+^ release rate (Ortenblad et al., 2011; Gejl et al., 2014) and impaired Ca^2+^ uptake caused by reduced Ca^2+^ ATPase (SERCA) activity (Duhamel et al., 2006b; Nielsen et al., 2009) and compromised membrane excitability (Nielsen et al., 2009; Renaud et al., 2023) are possible explanations. Reduced muscle glycogen content has been associated with a compromised SR Ca^2+^ release rate and half-relaxation time, as measured *in vitro* in muscle homogenate preparations (Nielsen et al., 2009; Ortenblad et al., 2011). In humans, contractile measurements are affected by fatigue (Booth et al., 1997), and accelerated reductions in Ca^2+^ release and uptake rates have been observed when exercising with low compared to higher muscle glycogen levels, as determined using *in vitro* assays (Duhamel et al., 2006a). If similar *in vivo* impairments in SR function occur during intense contractions in humans, the consequences of reduced glycogen content during constant-load exercise may include exacerbated rates of force development (RFD) and relaxation. However, few studies have investigated the effects of experimentally manipulated low glycogen content on human muscle contractile features. Thus, the second aim of this study was to examine whether human muscle contractile function, determined by the maximal voluntary contraction (MVC), electrically induced single-twitch maximal force, RFD, and rate of relaxation, is affected by the glycogen content. We hypothesized that exercise- and diet-induced low initial muscle glycogen content would impair human muscle contractile function, as determined by the MVC, single-twitch maximal force, RFD, and rate of relaxation before and immediately after the one-legged exercises to task failure.

Regarding compromised membrane excitability, increased Na^+^/K^+^-ATPase (NKA) activity during contractile activity is a prerequisite for delaying extracellular K^+^ build-up and preserving excitability (Hostrup et al., 2021). When glycogen is utilized during contractions, protein kinase A (PKA) activation causes phosphorylation of several proteins, including phospholemman (FXYD1) at Ser^68^, which inhibits NKA in the unphosphorylated state (Crambert et al., 2002). This results in increased NKA activity and maximal Na^+^/K^+^ transport capacity (Bibert et al., 2008). Exercise with low glycogen content upregulates the 5′AMP-activated protein kinase (AMPK) activity (Bartlett et al., 2013), which in contrast stimulates NKA activity (Ingwersen et al., 2011; Benziane et al., 2012). With low glycogen levels, a reduction in ATP provision from glycogenolysis and glycolysis can be expected (Dutka and Lamb, 2007; Jensen et al., 2020; Gaitanos et al., 1993). Also, ATP concentrations per see must be expected to be severely reduced (Karatzaferi et al., 2001). Reduced ATP availability compromise NKA activity. Therefore, the net effect of low glycogen content on the regulation of NKA activity remains unclear. Thus, the third aim of this study was to determine the extent to which the muscle glycogen content alters phosphorylation of the NKA regulatory proteins AMPK and FXYD1. We hypothesized that low muscle glycogen content alter exercise-induced phosphorylation of the NKA regulatory proteins, AMPK and FXYD1 indicating lower NKA activity and attenuated ion handling.

We investigated these hypotheses in healthy young male participants who performed two different exercise tasks, with one leg in an exercise- and diet-induced glycogen-depleted state and one leg in a glycogen-loaded state. The first task was to perform 5 min of intense two-legged cycling, where muscle biopsies were taken at rest and after exercise to investigate the NKA protein abundance and phosphorylation of NKA regulatory proteins. The second task involved two sessions of one-legged knee extensor exercise continued until task failure, in order to access possible differences in exercise performance and muscle contractile characteristics at rest, task failure, and recovery between the glycogen-loaded and glycogen-depleted legs.

## 2 Materials and methods

### 2.1 Participants

Ten healthy males provided written informed consent to participate in this study. Studies assessing muscle characteristics in humans, especially those incorporating muscle biopsies, frequently operate with small sample sizes due to ethical constraints, invasive methodology, and the high level of detail captured per participant, with similar studies also using comparable participant numbers (n = 8–10) (Duhamel et al., 2006a; Benziane et al., 2011; Bartlett et al., 2013). Participants were recreationally active, with a mean ± SD age of 25 ± 2 years, height of 184 ± 7 cm, and body mass of 79.0 ± 10.1 kg. The study was approved by the Ethics Committee of the Copenhagen and Frederiksberg communities (KF 01-183/02) and conducted in accordance with the Code of Ethics of the World Medical Association (Declaration of Helsinki) at the Department of Nutrition, Exercise and Sports, University of Copenhagen, Denmark.

### 2.2 Peak oxygen uptake and familiarization trials

The cycling tests were conducted using a Monark 839E ergometer (Monark Exercise AB, Vansbro, Sweden). All participants completed incremental cycle exercises comprising 10 min at 90 W and 10 min at 160 W, followed by 25 W increments each minute until exhaustion, to determine the peak oxygen uptake ( $\dot{V}$ O_2peak_). Expired gas O_2_ and CO_2_ fractions and respiratory volumes were analyzed using a daily calibrated metabolic cart (Viasys Masterscreen CPX, Jaeger, Hoechberg, Germany). The $\dot{V}$ O_2peak_ was 4.18 ± 0.89 L min^–1^, which corresponded to 53.0 ± 12.1 mL O_2_·kg^–1^·min^–1^, and the peak heart rate (HR_peak_) was 195.1 ± 9.3 beats·min^–1^. Participants then completed a familiarization trial during which they cycled to exhaustion at a work rate corresponding to approximately 90% $\dot{V}$ O_2peak_. On a second day, the participants repeated this test, and the work rates were adjusted individually to ensure that the time to task failure (i.e., when participants were unable to sustain the initial power output) was approximately 5 min. This cycling trial was followed by familiarization trials for one-legged knee extensor exercise (Andersen et al., 1985), including electrical stimulation procedures to determine muscle contractile function (see details below). The work rate designed to elicit task failure in approximately 5 min, a similar duration as for the cycling trial, was determined through at least one trial with each leg and workload adjustments according to the performance from a starting workload of 60 W.

### 2.3 Glycogen depletion trials and dietary manipulations

The purpose of the first part of the experiment was to achieve one leg in a glycogen-loaded state and the other leg in a glycogen-depleted state prior to the experimental trials involving muscle biopsies and determination of exercise performance. The legs chosen to be glycogen-loaded or glycogen-depleted were randomized and counterbalanced.

**FIGURE 1:**
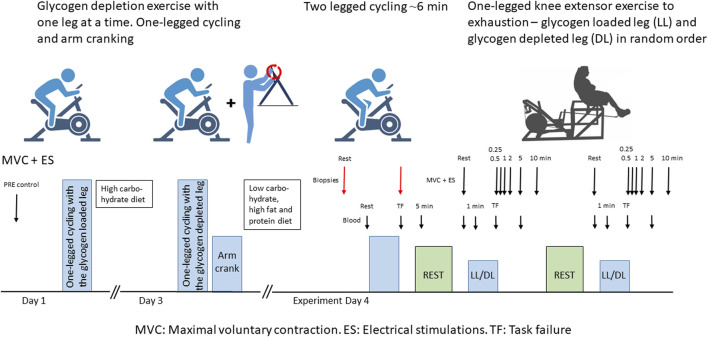
Schematic overview of the experimental setup. Red arrows indicate muscle biopsy sampling times, large black arrows indicate maximal voluntary contractions (MVC) and electrical stimulations (ES), and small black arrows indicate arm venous blood sampling times. LL: glycogen-loaded leg. DL: glycogen-depleted leg.

For the glycogen-loaded leg, the participants first completed a glycogen depletion exercise, followed by consuming a high-carbohydrate diet, based on a previous protocol (Pilegaard et al., 2002). Both continuous and intermittent one-legged cycling exercise were performed. The one-legged $\dot{V}$ O_2peak_ ( $\dot{V}$ O_2peak_ _1-leg_) was assumed to be equivalent to 74% of the two-legged cycling $\dot{V}$ O_2peak_ (Pernow and Saltin, 1971) and conducted with a counterbalance on the pedal crank. Participants started with a 6 min warm-up at 50 W, followed by 20 min of one-legged cycling at 60% $\dot{V}$ O_2peak 1-leg_ (114 ± 34 W). This was followed by repeated sessions of 90 s one-legged cycling (1:1 exercise/rest ratio), with an initial intensity of 90% $\dot{V}$ O_2peak 1-leg_ (175 ± 48 W). When participants could not complete 90 s of cycling, the work rate was decreased to 85% $\dot{V}$ O_2peak 1-leg_ for subsequent sessions. When the participant could not sustain the 85% $\dot{V}$ O_2peak 1-leg_, the work rate was successively reduced to 75%, 70%, and 65% $\dot{V}$ O_2peak 1-leg_, with 90–180 s of rest between the work rate intervals. In total, 63 ± 12 intervals were completed. A final one-legged exercise session to exhaustion was then conducted at 85% $\dot{V}$ O_2peak 1-leg_ (duration: 112 ± 77 s at 163 ± 44 W). Immediately after the first glycogen depletion one-legged cycling exercise, participants consumed a high-carbohydrate diet (78%, 13%, and 9% of the energy from carbohydrates, fat, and protein, respectively) lasting 2 days, to achieve glycogen super-compensation. An overview of the experimental design is shown in Figure 1.

On the evening of day 3, participants completed a similar glycogen depletion protocol by exercising one leg (the glycogen-depleted leg) with minor workload adjustments. Participants started with a 6 min warm-up at 50 W, followed by 20 min of one-legged cycling at 60% $\dot{V}$ O_2peak 1-leg_ (120 ± 30 W). This was followed by repeated sessions of 90 s of one-legged cycling (1:1 exercise/rest ratio), with an initial intensity of 90% $\dot{V}$ O_2peak 1-leg_ (180 ± 44 W). When participants were unable to complete 90 s at 90% $\dot{V}$ O_2peak 1-leg_, the work rate was decreased to 85% $\dot{V}$ O_2peak 1-leg_ for subsequent sessions. When 85% $\dot{V}$ O_2peak 1-leg_ could not be sustained, the work rate was successively reduced to 75%, 70%, and 65% $\dot{V}$ O_2peak 1-leg_, with 90–180 s of rest between the work rate intervals. In total, 66 ± 22 of the 90 s intervals were completed. A final one-legged exercise session to exhaustion was then performed at 85% $\dot{V}$ O_2peak 1-leg_ (duration: 73 ± 19 s at 166 ± 43 W). To achieve the highest possible glycogen depletion in the glycogen-depleted leg, the final session was after 15 min of rest followed by an additional arm-cranking exercise at 82 ± 24 W for 28 ± 5 min, to elicit further decreases in liver glycogen levels. After completion of the one-legged glycogen depletion trial, participants consumed a low-carbohydrate, high-fat, and high-protein diet overnight (4.3 MJ and less than 22 g of carbohydrate) and at breakfast (2.7 MJ and less than 22 g of carbohydrate) (Pilegaard et al., 2002), to ensure that muscle glycogen levels remained low for the experimental trials the following morning.

### 2.4 Exercise trials, muscle function determination, and blood sampling

#### 2.4.1 Two-legged cycling

To determine both contractile characteristics with MVC and electrical stimulation as well as protein phosphorylation as close to task failure as possible, we performed two different exercise trials on the experimental day. First, participants completed a two-legged cycling session leading to task failure (i.e., participants were unable to sustain the initial power output) in 367 ± 111 s (Figure 1). The initial power output (288 ± 61 W) was set at approximately 90%–105% $\dot{V}$ O_2peak_; if task failure was anticipated within the first minutes, the work rate was decreased to ensure continuation of the trial for at least 4 min (mean power output: 274 ± 43 W). This procedure ensured that the maximal recruitment of all fibers occurred early in the exercise session and that task failure was achieved in the required time frame of approximately 5 min. Before the two-legged cycling exercise trial, a catheter was inserted into the antecubital vein (due to technological issues, we did not catheterize three subjects, n = 7). Blood samples were collected before, immediately after, and after 300 s of recovery from the exercise session and immediately analyzed for plasma pH and concentrations of K^+^, Na^+^, lactate, and glucose (ABL 700, Radiometer, Copenhagen, Denmark).

#### 2.4.2 One-legged knee extensor exercise

After 2 hours of rest with *ad libitum* water intake but no food ingestion, participants sat in a seat with one foot strapped in a custom boot attached to a one-legged knee extensor ergometer (Andersen et al., 1985). Participants performed a one-legged knee extensor exercise (63 ± 7 W) to task failure, when the RPM decreased from 60 to 45, with one leg. After 45 min of rest, the protocol was repeated with the other leg, in a randomized and counterbalanced order. The quadriceps MVC was determined before exercise as the highest of three 3–5 s maximal contractions, each separated by 30 s, and in recovery as single maximal contractions at 15, 30, 60, 120, 300, and 600 s post-exercise. For MVC measurements, the knee was fixed at an angle of 90°. The force was recorded using a calibrated strain gauge (Model 615; Tedea-Huntleigh Electronics, UK) attached to a custom boot, sampled at 1 kHz, and reported in Newtons (N). Immediately after the MVC was determined, at approximately 1 s, double-pulse electrical stimulation (400 V, approximately 100 mA) was delivered (2 × 1 ms separated by 10 ms, corresponding to 100 Hz) using a Digitimer DS7AH and DG2A generator (Digitimer, Hertfordshire, UK) and 5 × 9 cm^2^ electrodes (PALS platinum, Axelgaard, Lystrup, Denmark) placed over the belly of the rectus femoris. Prior to the experiment, the optimal stimulation intensity was determined by increasing the stimulation current until the twitch response plateaued, and 110% of this intensity was used in all experiments. Blood samples were collected before, during (60 s), immediately after, and 300 s during recovery from the high-intensity exercise bouts and immediately analyzed for plasma pH, [K^+^], [Na^+^], [lactate], and [glucose] (ABL 700, Radiometer, Copenhagen, Denmark).

### 2.5 Muscle biopsies

Under local anesthesia induced by xylocaine injections (2 mL of lidocaine without epinephrine, xylocaine 20 mg/mL; Astra Zeneca, Cambridge, UK), two incisions were made in the vastus lateralis of each leg. Four biopsies were taken from each participant: one in the glycogen-depleted leg and one in the glycogen-loaded leg before the two-legged cycling exercise (before exercise) and one in each leg immediately (first biopsy: 18 ± 8 s, second biopsy: 43 ± 9 s, depleted leg: 33 ± 17 s, loaded leg: 27 ± 14 s) after exercise (after exercise) (Figure 1). Biopsy samples were rapidly frozen in liquid nitrogen. Most of the rapidly frozen muscle tissue was freeze-dried and dissected free from blood, fat, and connective tissue before further analysis; approximately 20 mg was stored as whole muscle tissue for NKA content determination. The analyses of the muscle tissue were prioritized in the following order [protein abundance and phosphorylation (n = 10), glycogen content (n = 8–9), NKA content (n = 7–8) and muscle pH (n = 5–7)] and due to limited amount of tissue in some biopsies n differs between the obtained data.

#### 2.5.1 Glycogen and pH content

Muscle glycogen content was determined in muscle samples using a modified spectrophotometric method (Harris et al., 1974). First, 1–2 mg_d.w._ of the freeze-dried muscle tissue samples were extracted in 0.5 mL 1 M HCl and hydrolyzed at 100°C for 3 h. Then, the glucose content was determined by the hexokinase method using a glucose kit and a PentraC 400 analyzer (TrioLab). The amount of NADPH formed was directly proportional to the glucose content and was measured spectrophotometrically at 340 nm. In addition, the muscle pH was measured using a small glass electrode (XC 161, Radiometer-analytical, France) after homogenization of approximately 1 mg_d.w._ of the samples in 100 µL non-buffered solution containing 145 mM KCl, 10 mM NaCl, and 5 mM sodium fluoride (Mannion et al., 1993).

#### 2.5.2 Western blotting

The protein abundance and phosphorylation status were determined, as previously described by our laboratory (Thomassen et al., 2010). Briefly, samples of freeze-dried muscle tissue were homogenized (Qiagen Tissuelyser II, Retsch GmbH, Haan Germany) in fresh buffer containing 10% glycerol, 20 mM Na-pyrophosphate, 150 mM NaCl, 50 mM HEPES (pH 7.5), 1% NP-40, 20 mM β-glycerophosphate, 2 mM Na_3_VO_4_, 10 mM NaF, 2 mM PMSF, 1 mM EDTA (pH 8), 1 mM EGTA (pH 8), 10 μg mL^–1^ aprotinin, 10 μg mL^–1^ leupeptin, and 3 mM benzamidine. Next, samples underwent end-over-end rotation for 1 h at 4°C and were centrifuged at 16,500 g for 30 min at 4°C; the supernatant (lysate) was used for further analyses. The total protein concentration in each sample was determined using a BCA standard kit (Pierce), and samples were mixed with 6× Laemmli buffer (7 mL 0.5 M Tris-base, 3 mL glycerol, 0.93 g DTT, 1 g SDS, and 1.2 mg bromophenol blue).

Equal amounts of total protein were loaded into each well of a precast gel (Bio-Rad Laboratories, USA). Two protein markers on each gel (all blue and dual colors; Bio-Rad Laboratories, USA) were used to determine the molecular weights of the proteins quantified. Proteins were separated according to their molecular weights by SDS-PAGE and semi-dry transferred to a PVDF membrane (Millipore A/S, Denmark). The membranes were blocked in either 2% skimmed milk or 3% BSA in Tris-buffered saline including 0.1% Tween-20 (TBST) before an overnight incubation with the primary antibody at 4°C. The membranes were washed in TBST and incubated for 1 h at room temperature with horseradish peroxidase (HRP)-conjugated secondary antibody. Membranes were then washed three times for 15 min each in TBST. The bands were visualized with ECL (Millipore) and recorded using a digital camera (Kodak Image Station 2000MM, Kodak, Denmark). The band intensities were quantified using 1D software (Kodak, Denmark) and were determined as the total band intensity minus the background intensity. Individual values were normalized to the mean values of the resting glycogen-loaded leg samples.

#### 2.5.3 Antibodies

The primary antibodies used in the present experiment were optimized using mixed human standard lysates, to ensure that the amount of protein loaded would result in band signal intensities localized to the steep, linear part of the standard curve. To determine changes in abundance of proteins involved in Na^+^ and K^+^ muscle cell homeostasis, the following antibodies and corresponding migration of the quantified signal were used: NKAα_1_: 100 kDa, C464.6 (Millipore); NKAα_2_: 100 kDa, 07-647 (Upstate); NKAβ_1_: 40–50 kDa, MA3-930 (Affinity Bioreagents); N-terminal FXYD1: 12 kDa, FXYD1 (kindly donated by M. Shattock, (Fuller et al., 2004); and Na^+^/H^+^ exchanger (NHE1): 110 kDa, MAB3140 (Chemicon).

Alterations in the phosphorylation status were determined using the following antibodies: acetyl-CoA carboxylase (ACC) βser^221^ phosphorylation: 260 kDa, phospho-specific ACCαser^79^ antibody (#07-303, Millipore); α-AMPKthr^172^ phosphorylation: 62 kDa, #2531 (Cell Signaling Technology); unspecific FXYD1 phosphorylation: 12 kDa, AB_FXYD1 (kindly donated by Dr. J. Randall Moorman, University of Virginia); FXYD1ser^68^ phosphorylation: 12 kDa, AB_FXYD1ser68 (kindly donated by Dr. D. Bers, Loyola University); FXYD1ser^63^ phosphorylation: 12 kDa, #2261 (Cell Signaling Technology); and combined FXYD1ser^68^ and thr^69^ phosphorylation, 12 kDa, #9621 (Cell Signaling Technology). HRP-conjugated goat anti-mouse and goat anti-rabbit (P-0447 and P-0448, respectively; DAKO, Denmark) secondary antibodies were used.

#### 2.5.4 Antibody specificity

The phospho-specific ACCαser^79^ antibody (#07-303, Millipore) recognizes the equivalent ser^221^ in human ACCβ (Wojtaszewski et al., 2003), and ACCβser^221^ phosphorylation is a downstream target of AMPK and well-known indicator of AMPK activity (Birk and Wojtaszewski, 2006).

**FIGURE 2:**
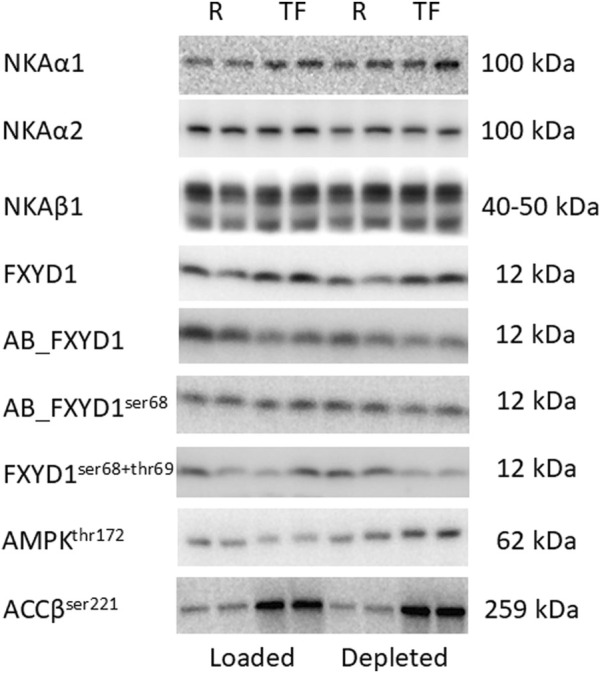
Representative western blots shown for one subject in duplicates. Muscle biopsy tissue was divided into two tubes after freeze drying and dissection, thereafter, treated separately with the same protocol to reduce method variation. The band migration is indicated at the right side. R: Rest and TF: Task failure. NKA: Na^+^/K^+^-ATPase. FXYD1: phospholemman. AMPK: 5′AMP-activated protein kinase. ACC: acetyl-CoA carboxylase.

FXYD1 has three known phosphorylation sites (ser63, ser68, and thr69) located at its C-terminus (Bibert et al., 2008; Fuller et al., 2009). The polyclonal AB_FXYD1 antibody recognizes total unphosphorylated FXYD1 proteins with a 16 amino acid epitope at the C-terminus. Thus, the more phosphorylated FXYD1 proteins and the more phosphorylated FXYD1 sites, the lower the binding of AB_FXYD1. Therefore, the level of nonspecific FXYD1 phosphorylation was determined as AB_FXYD1/N-terminus total FXYD1, where a decrease in the AB_FXYD1 signal intensity represented an increase in nonspecific phosphorylation (Thomassen et al., 2011). FXYD1 phosphorylation on ser^68^ was first determined as (AB_FXYD1ser68/FXYD1) and then normalized to the nonspecific FXYD1 phosphorylated content determined by AB_FXYD1 (AB_FXYD1ser68/AB_FXYD1), to consider the concomitant phosphorylation of ser^63^ and thr^69^ (Thomassen et al., 2016). Furthermore, FXYD1ser^63^ phosphorylation and combined FXYD1ser^68^ and FXYD1thr^69^ phosphorylation were determined using motif- and phospho-specific antibodies #2261 and #9621, respectively, which have shown specificity for FXYD1 at approximately 12 kDa (Thomassen et al., 2011). Representative western blots are shown in Figure 2.

#### 2.5.5 [^3^H]-ouabain binding site content

To determine the total NKA content, the [^3^H]-ouabain binding site content was measured in the muscles before and after exercise in both glycogen-loaded and glycogen-depleted legs. Approximately 20 mg of whole muscle was used to measure the [^3^H]-ouabain binding site content, as previously described (Norgaard et al., 1984; Petersen et al., 2012). Briefly, each sample was washed twice for 10 min at 37°C in vanadate buffer (250 mM sucrose, 10 mM Tris-HCl, 3 mM MgSO_4_, and 1 mM NaVO_4_; pH 7.3). Following washing, samples were incubated for 2 h at 37°C in vanadate buffer with the addition of [^3^H]-ouabain (2.0 μCi mL^-1^ and 10^-6^ M, PerkinElmer Inc., Boston, MA, USA). The muscle was then placed in ice-cold vanadate solution for 4 × 30 min to remove any unbound [^3^H]-ouabain. Muscle samples were then blotted on filter paper and weighed before soaking in 500 μL 5% trichloroacetic acid and 0.1 mM ouabain for approximately 20 h. Then, 2.5 mL of a scintillation cocktail (Ultima Gold, Packard, PerkinElmer Inc., Boston, MA, USA) was added before the liquid scintillation counting of [^3^H]-ouabain. The [^3^H]-ouabain binding site content was calculated using the sample wet weight and specific activity of the incubation buffer and samples and was expressed as pmol·g_w.w._^−1^. The final [^3^H]-ouabain content was then calculated, accounting for nonspecific binding, using a correction factor for [^3^H]-ouabain impurities, loss of bound [^3^H]-ouabain during washout, and incomplete saturation, as previously described (Norgaard et al., 1984).

### 2.6 Statistics

All statistical analyses were performed using SPSS (version 27; IBM Corp., Armonk, NY, US). The data were inspected for normality using Q-Q plots. Differences in one-legged high-intensity exercise performance were tested using a paired t-test. Changes in arm venous plasma variables before and after the two-legged cycling exercise were tested using one-way ANOVA with SIDAK-adjusted *post hoc* analysis. To estimate the differences in all other variables between treatment (glycogen-loaded vs. glycogen-depleted) and time (rest vs. task failure or rest, exercise, and recovery), a two-factor linear mixed model was used, with the glycogen content and time as fixed effects and a random effect for subjects. If a main effect or interaction was observed, a SIDAK-adjusted *post hoc* analysis was performed to determine specific differences. Data are presented as mean ± SD. *P*-values < 0.05 indicated statistical significance. Exact *P*-values were only shown if *P* < 0.10 and *P* > 0.05.

## 3 Results

### 3.1 Two-legged cycling

#### 3.1.1 Muscle glycogen content

**FIGURE 3:**
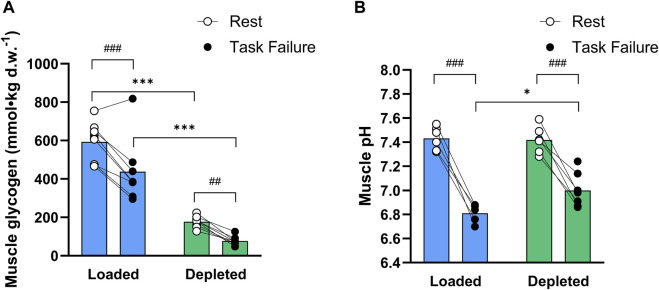
**(A)** Total muscle glycogen content (mmol kg_d.w._^−1^) in the glycogen-loaded and glycogen-depleted legs at rest and task failure from two-legged intense exercise cycling (n = 9, pre-exercise; n = 8, post-exercise). **(B)** Muscle pH values in the glycogen-loaded and glycogen-depleted legs at rest (n = 6) and task failure (n = 5 and n = 7, respectively). Data are reported as means, with individual values marked as white (rest) and black circles (task failure). There was a main effect of exercise (*P* < 0.001) for both the muscle glycogen content and pH, a main effect of treatment (*P* < 0.001) for the muscle glycogen content, and a main tendency of treatment (*P* = 0.075) and interaction (*P* = 0.065) for the muscle pH. ^###^(*P* < 0.001) and ^##^(*P* < 0.01) indicate a significant difference at rest and task failure from the intense exercise session in the same leg. *Indicates a significant difference between the glycogen-loaded and glycogen-depleted legs at task failure (*P* < 0.05).

Muscle glycogen content decreased (main effect of exercise, *P* < 0.001) during the two-legged cycling exercise and was higher (main effect of treatment, *P* < 0.001) in the glycogen-loaded leg than in the glycogen-depleted leg, both at rest (593 ± 100 vs. 177 ± 29 mmol·kg_d.w._^−1^, respectively; *P* < 0.001, n = 9) and at task failure (438 ± 166 vs. 77 ± 23 mmol·kg_d.w._^−1^, respectively; *P* < 0.001, n = 8) in the two-legged cycling exercise (Figure 3A). The absolute reduction in glycogen content was not significantly different between legs (152 ± 100 vs. 98 ± 31 mmolkg_d.w._^−1^; *P* = 0.23, n = 8).

#### 3.1.2 Muscle homogenate pH

The muscle homogenate pH decreased during the two-legged cycling exercise (main effect of exercise, *P* < 0.001) in the glycogen-loaded leg, from 7.43 ± 0.10 (n = 6) to 6.81 ± 0.08 (n = 5, *P* < 0.001), and in the glycogen-depleted leg, from 7.42 ± 0.11 (n = 6) to 7.00 ± 0.14 (n = 7, *P* < 0.001). The muscle homogenate pH was lower (main tendency of treatment, *P* = 0.075; interaction, *P* = 0.065) in the glycogen-loaded than in the glycogen-depleted leg at task failure (*P* < 0.05) (Figure 3B).

#### 3.1.3 Arm venous plasma variables

The two-legged cycling exercise induced the following respective changes in arm venous plasma values from rest (n = 7) to task failure and 300 s after exercise (n = 5): [lactate] increased from 0.77 ± 0.14 to 7.86 ± 3.43 mM (*P* < 0.001) and further to 13.24 ± 0.72 mM (*P* < 0.01); plasma [K^+^] increased from 3.63 ± 0.19 to 4.12 ± 0.44 mM (*P* < 0.05) and decreased after exercise to 3.28 ± 0.2 mM (*P* < 0.01); [Na^+^] increased from 138.9 ± 1.1 to 145.8 ± 3.1 mM (*P* < 0.001) and decreased after exercise to 142.4 ± 1.1 mM (*P* < 0.05); and pH declined from 7.38 ± 0.01 to 7.23 ± 0.07 and 7.19 ± 0.02 (*P* < 0.001). The data from the one-legged exercise are shown below.

#### 3.1.4 Muscle protein abundances

**FIGURE 4:**
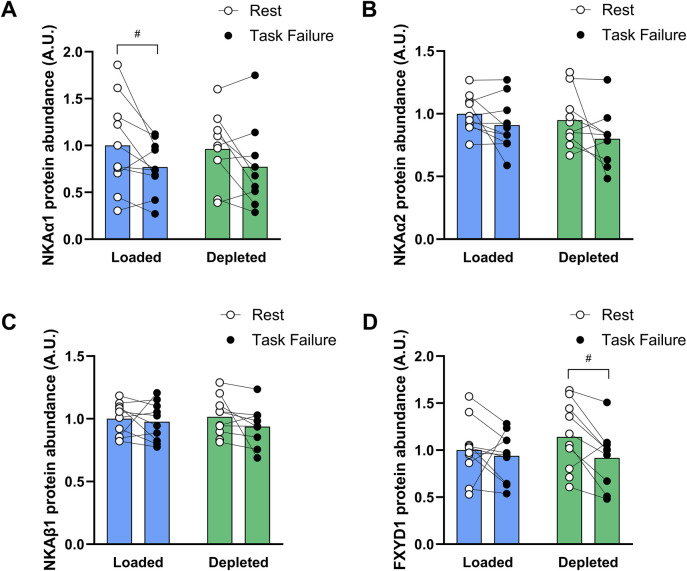
Protein abundance of the NKA α_1_ **(A)**, α_2_ **(B)**, β_1_ **(C)** isoforms and FXYD1 **(D)** in the glycogen-loaded and glycogen-depleted legs at rest and task failure from two-legged intense exercise cycling. Data are normalized to the mean rest value in the glycogen-loaded leg and reported in arbitrary units as means (n = 10), with individual values marked as white (rest) and black circles (task failure). There was a main effect of exercise (*P* < 0.05) for NKAα1, NKAα2, and FXYD1. ^#^Indicates a significant difference at rest versus task failure from the intense exercise session in the same leg (*P* < 0.05).

NKAα_1_- and α_2_-subunit isoform protein abundances decreased after exercise compared to those before the two-legged cycling exercise (main effect of exercise, *P* < 0.05), and the NKAα1 abundance was lower in the glycogen loaded leg at task failure than at rest (*P* < 0.05). Both NKAα1 and NKAα2 abundances in the depleted leg tended (*P* = 0.091 and *P* = 0.060, respectively) to be lower at task failure than at rest (n = 10; Figures 4A,B). The NKAβ_1_-subunit isoform protein abundance was unchanged with exercise and was not different in the glycogen-loaded and glycogen-depleted legs (n = 10, Figure 4C). The FXYD1 abundance was reduced at task failure from exercise compared to that at rest (main effect of exercise, *P* < 0.05) and was lower in the depleted leg (*P* < 0.05) after exercise than at rest; however, there was no difference between the legs (n = 10, Figure 4D). The NHE1 abundance was unaffected by exercise, while glycogen depletion induced a higher NHE1 abundance (main effect of treatment, *P* < 0.01), which was not significant at rest or task failure (n = 10; data not shown).

The total NKA content, determined by the [^3^H]-ouabain binding site content, was not different at rest and task failure and was not different between the glycogen-loaded and glycogen-depleted legs at rest (310 ± 66 vs. 337 ± 47 pmol·g_w.w._^−1^; n = 8) or task failure (337 ± 64 vs. 338 ± 56 pmol·g_w.w._^−1^; n = 7 and n = 8, respectively).

#### 3.1.5 Protein phosphorylation

**FIGURE 5:**
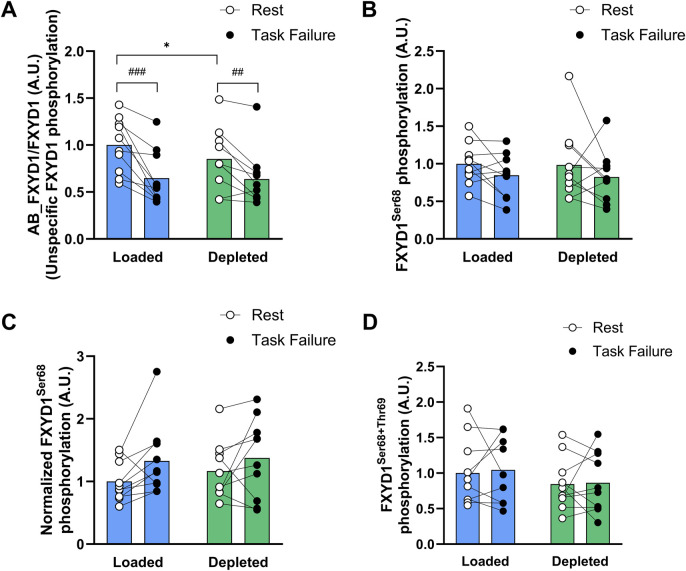
Nonspecific and specific FXYD1 phosphorylation levels in the glycogen-loaded and glycogen-depleted legs at rest and task failure from two-legged intense exercise cycling. **(A)** AB_FXYD1/FXYD1 determines the nonspecific FXYD1 phosphorylation normalized to the total FXYD1 abundance (see methods). A decrease in the AB_FXYD1 signal intensity represents an increase in nonspecific phosphorylation (Thomassen et al., 2011). **(B)** FXYD1ser^68^ phosphorylation normalized to total FXYD1 abundance. **(C)** Normalized FXYD1ser^68^ phosphorylation determines the FXYD1ser^68^ phosphorylation by considering concomitant phosphorylation on FXYD1ser^63^ and thr^69^ by normalizing to the AB_FXYD1 signal intensity (Thomassen et al., 2016). **(D)** FXYD1ser^68^+thr^69^ phosphorylation normalized to total FXYD1 abundance. Data are normalized to mean values at rest in the glycogen-loaded leg and reported in arbitrary units as means (n = 10), with individual values marked as white (rest) and black circles (task failure). There was a main effect of exercise for nonspecific FXYD1 phosphorylation (*P* < 0.001) and normalized FXYD1ser^68^ phosphorylation (*P* < 0.05) and a main effect of treatment (*P* < 0.05) for combined FXYD1ser^68^+thr^69^ phosphorylation and a main tendency of treatment (*P* = 0.064) for nonspecific FXYD1 phosphorylation. ^###^(*P* < 0.001) and ^##^(*P* < 0.01) indicate a significant difference between rest and task failure from exercise in the same leg. *Indicates a significant difference between the glycogen-depleted and glycogen-loaded legs at rest (*P* < 0.05).

Nonspecific FXYD1 phosphorylation increased with exercise (main effect of exercise, *P* < 0.001) and was increased at task failure compared to that at rest in both the glycogen-loaded (*P* < 0.001) and glycogen-depleted legs (*P* < 0.01; Figure 5A). Furthermore, nonspecific FXYD1 phosphorylation tended (main tendency of treatment, *P* = 0.064) to be higher in the depleted legs than in the loaded legs. At rest, the level of nonspecific FXYD1 phosphorylation was greater (*P* < 0.05) in the glycogen-depleted legs than in the glycogen-loaded legs, while no difference was observed at task failure between the legs (n = 10; Figure 5A).

The FXYD1 phosphorylation at ser^68^ was unaltered by exercise or glycogen intervention (n = 10; Figure 5B). When considering concomitant phosphorylation at ser^63^ and thr^69^ (see Section 2.5.4), FXYD1ser^68^ phosphorylation increased (main effect of exercise, *P* < 0.05) during exercise and tended to be higher (*P* = 0.055) in the loaded leg at task failure but was unaffected by the glycogen level (n = 10; Figure 5C).

The combined FXYD1ser^68^ and FXYD1thr^69^ phosphorylation was unaffected by exercise and was higher (main effect of treatment, *P* < 0.05) in the glycogen-loaded leg than in the depleted leg but not at rest or task failure (n = 10; Figure 5D). The FXYD1ser^63^ phosphorylation remained constant during exercise and was unaffected by glycogen levels (data not shown).

**FIGURE 6:**
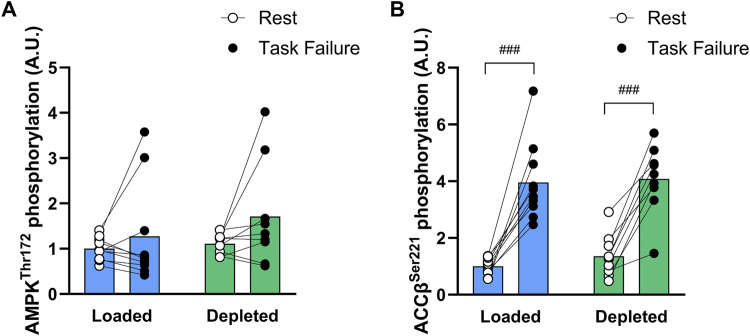
AMPK thr^172^ **(A)** and ACCβ ser^221^ **(B)** phosphorylation levels in the glycogen-loaded and glycogen-depleted legs at rest and task failure from two-legged intense exercise cycling. Data are normalized to mean values at rest in the glycogen-loaded leg and reported in arbitrary units as means (n = 10), with individual values marked as white (rest) and black circles (task failure). There was a main effect of exercise (*P* < 0.001) for ACCβ ser^221^ phosphorylation and a main effect of treatment (*P* < 0.05) and main tendency of exercise (*P* = 0.071) for AMPK thr^172^ phosphorylation. ^###^Indicates a significant difference between rest and task failure from intense exercise in the same leg (*P* < 0.001).

AMPKthr^172^ phosphorylation tended to be increased by exercise (main tendency of exercise, *P* = 0.071) and tended to be higher (*P* = 0.084) at task failure in the depleted leg than that at rest (Figure 6A). The AMPKthr^172^ phosphorylation was higher (main effect of treatment, *P* < 0.05) in the glycogen-depleted leg than in the loaded leg but not before or after exercise (n = 10; Figure 6A). The ACCβser^221^ phosphorylation increased with exercise (main effect of exercise, *P* < 0.001) and was higher at task failure in both the glycogen-loaded (*P* < 0.001) and glycogen-depleted (*P* < 0.001) legs. The glycogen level had no effect on the ACCβser^221^ phosphorylation (n = 10; Figure 6B).

### 3.2 One-legged knee extensor exercise to exhaustion

#### 3.2.1 Performance

**FIGURE 7:**
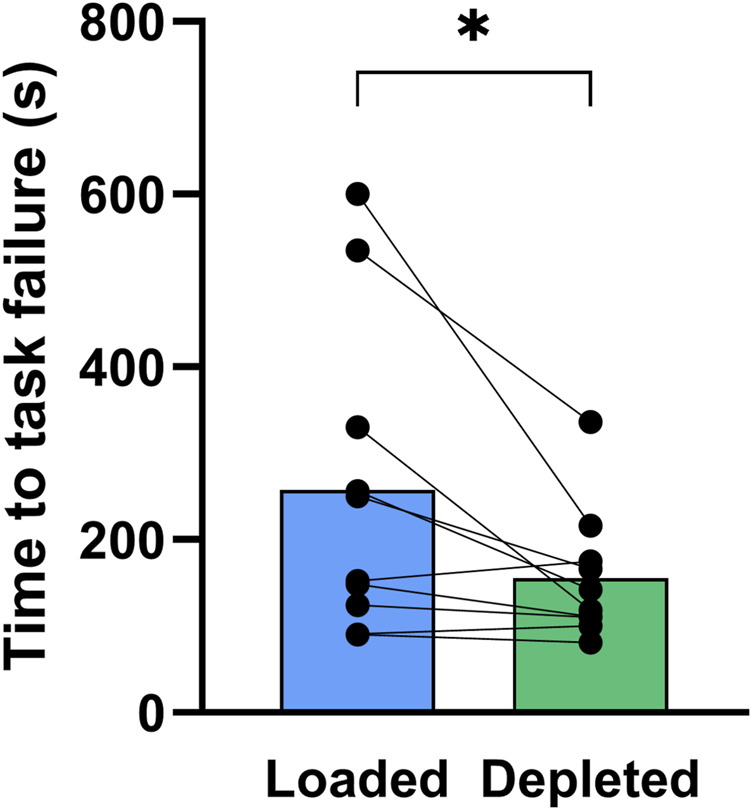
Time to task failure in the one-legged knee extensor exercise performed with the glycogen-loaded legs (blue) and the glycogen-depleted legs (green). Data are shown as means with individual values marked as black circles. *Indicates a significant difference between the glycogen-loaded and glycogen-depleted legs (*P* < 0.05).

Time to task failure in the one-legged knee extensor exercise was 65% longer (*P* < 0.05) in glycogen-loaded legs than in glycogen-depleted legs (258 ± 181 vs. 156 ± 75 s; n = 10; Figure 7).

#### 3.2.2 MVC

The quadriceps MVC was similar between the glycogen-loaded and glycogen-depleted legs, both at rest and task failure (Table 1). Specifically, the MVC was reduced (main effect of time, *P* < 0.001) to approximately 62% (*P* < 0.01), 75% (*P* < 0.01), and 80% (*P* < 0.05) at 15, 30, and 60 s of recovery, respectively. The MVC tended (main tendency for treatment, *P* = 0.06) to be higher (*P* = 0.08) in the glycogen-loaded leg than in the glycogen-depleted leg after 15 s of recovery.

**TABLE 1:** Quadriceps MVC pre and post dynamic one-legged knee extensor exercise to task failure in glycogen-depleted and glycogen-loaded conditions.

Condition	Pre exercise	Time after exercise to task failure (s)					
		15	30	60	120	300	600
Glycogen-loaded leg (N)	523 ± 123	382 ± 49^##^	384 ± 73^##^	408 ± 68^#^	424 ± 56	448 ± 54	483 ± 71
Glycogen-depleted leg (N)	499 ± 126	310 ± 83^##^	370 ± 72^##^	386 ± 65^#^	406 ± 70	442 ± 76	464 ± 70
Glycogen-loaded leg (% of pre exercise)		66 ± 13^$^	75 ± 11^##^	80 ± 10^#^	84 ± 13	89 ± 17	95 ± 17
Glycogen-depleted leg (% of pre exercise)		59 ± 10^$^	76 ± 13^##^	80 ± 12^#^	83 ± 11	91 ± 12	96 ± 16

Maximal voluntary contraction was determined at rest (pre exercise) and post dynamic one-legged knee extensor exercise to task failure in each of the glycogen-loaded and glycogen-depleted legs. Data are given in absolute units and for measurements in recovery also expressed as percent of resting MVC before exercise. Data are expressed as means ± SD; n = 10, except for MVC at 15 s, where n = 8 in the glycogen-depleted leg and n = 5 in the glycogen-loaded leg. ^#^(*P* < 0.05), ^##^(*P* < 0.01) indicates significant difference from pre exercise. ^$^Indicates a tendency (*P* < 0.10) for a difference between the loaded and depleted leg.

#### 3.2.3 Electrically induced isometric twitch before and after dynamic one-legged knee extensor exercise

The quadriceps electrically induced maximal twitch force was similar at rest in the glycogen-loaded and glycogen-depleted legs (114 ± 40 vs. 112 ± 32 N, respectively; n = 10) and was reduced (main effect of time, *P* < 0.001) at 30 s after exercise (*P* < 0.001) in both legs to 60%–65% of the resting single-twitch maximal force. A main effect of treatment (*P* < 0.05) was evident for the single-twitch maximal force, which was greater in the depleted leg than in the loaded leg, but with no detectable time-specific differences between the glycogen-loaded and glycogen-depleted legs (Table 2).

**TABLE 2:** Muscle contraction characteristics in recovery from dynamic one-legged knee extensor exercise to task failure in glycogen-depleted and glycogen-loaded conditions.

Muscle contraction characteristics	Condition	Time after exercise to task failure (s)				
		30	60	120	300	600
Single-twitch maximal force (% of pre)^§§§^	Loaded	60.1 ± 9.7	62.8 ± 10.3	73.3 ± 17.4	72.5 ± 18.7	67.8 ± 15.9
	Depleted^†^	64.8 ± 9.3	70.8 ± 9.0	75.3 ± 9.7	76.4 ± 12.1	74.2 ± 8.3
RFD (% of pre)^§§§^	Loaded	58.5 ± 8.3	62.0 ± 9.5	73.4 ± 15.5	73.4 ± 17.3	68.7 ± 15.2
	Depleted^††^	63.6 ± 10.5	70.2 ± 11.2*	75.8 ± 11.6	78.1 ± 12.5	76.6 ± 10.3
Rate of relaxation (% of pre)^§§§^	Loaded	45.2 ± 11.5	50.8 ± 12.0	65.9 ± 17.5	73.5 ± 22.3	77.9 ± 24.1
	Depleted^††^	55.1 ± 11.2	64.0 ± 11.8*	73.0 ± 14.1	85.0 ± 14.7	87.5 ± 22.0

Electrically induced single-twitch maximal force, rate of force development (RFD) and rate of relaxation were determined at rest (pre exercise) and post dynamic one-legged knee extensor exercise to task failure in each of the glycogen-loaded and glycogen-depleted legs. Data are post-exercise values expressed as a percentage of pre-exercise in that condition (means ± SD). N = 10 for all measures. ^†^Indicates a main effect of treatment (*P* < 0.05). ^††^Indicates a main effect of treatment (*P* < 0.01). *Indicates significant difference from the glycogen-loaded leg (*P* < 0.05). ^§§§^Indicates a main effect of time (*P* < 0.001).

The RFD, determined from the electrically induced twitch, was similar in the glycogen-loaded and glycogen-depleted legs at rest (1,448 ± 542 vs. 1,417 ± 455 N/s, respectively; n = 10). The RFD was reduced (main effect of time, *P* < 0.001) at 30 s after exercise (*P* < 0.001) to 59%–64% of resting values (Table 2). The main effect of the treatment (*P* < 0.01) was evident as a higher RFD in the glycogen-depleted leg than in the glycogen-loaded leg, with a significant difference between legs detected 60 s post-exercise (*P* < 0.05; Table 2).

The rate of relaxation, determined from the electrically induced twitch, was similar in the glycogen-loaded and glycogen-depleted legs at rest (−787 ± 296 vs. −771 ± 204 N/s; n = 10). The rate of relaxation was markedly reduced (main effect of time, *P* < 0.001) at 30 s after exercise (*P* < 0.001) to 45%–55% of resting values (Table 2). A main effect of treatment (*P* < 0.01) and an interaction between treatment and time (*P* < 0.05) were evident as a lower rate of relaxation in the glycogen-loaded leg than in the glycogen-depleted leg, with a significant difference between the legs at 60 s post-exercise (*P* < 0.05; Table 2).

#### 3.2.4 Arm venous plasma concentrations and pH

The arm venous plasma [lactate] increased (main effect of time, *P* < 0.001) during the one-legged knee extensor exercise and was higher at task failure and recovery than before exercise in both legs. The arm venous plasma [lactate] was higher (main effect of treatment, *P* < 0.05) at task failure and recovery (*P* < 0.05) in the glycogen-loaded leg than in the glycogen-depleted leg (Table 3). The plasma [K^+^] was higher (main effect of time, *P* < 0.001) at task failure than before exercise (60 s) and during recovery from the one-legged knee extensor exercise in the glycogen-loaded (*P* < 0.05) and glycogen-depleted legs (*P* < 0.001), with no differences between treatments (Table 3). The arm-venous plasma [Na^+^] was higher (main effect of time, *P* < 0.001) at task failure than at rest and after 60 s (*P* < 0.05) of the one-legged knee extensor exercise, with no differences between treatments (Table 3). The arm venous pH decreased (main effect of time, *P* < 0.001) at 300 s of recovery from the one-legged knee extensor exercise compared to that at rest in both the glycogen-loaded (*P* < 0.05) and glycogen-depleted legs (*P* < 0.01) (Table 3). The arm venous [glucose] was similar before, during, and after the one-legged knee extensor exercise in both the glycogen-loaded and glycogen-depleted legs (Table 3).

**TABLE 3:** Arm venous plasma electrolyte, glucose concentrations and pH before, during and after one-legged knee extensor exercise to task failure in glycogen-depleted and glycogen-loaded conditions.

Variable (unit)	Condition	Time			
		Pre exercise	60 s	Task failure	300 s recovery
Lactate (mmol·l^–1^)^§§§^	Loaded	1.2 ± 0.3	1.4 ± 0.3	3.8 ± 1.5^###^	4.9 ± 1.0^###^
	Depleted^†^	1.3 ± 0.3	1.5 ± 0.4	2.6 ± 0.7^#^*	4.0 ± 1.7^###^*
K^+^ (mmol·l^–1^)^§§§^	Loaded	3.7 ± 0.3	3.8 ± 0.3	4.1 ± 0.3^‡^	3.7 ± 0.2
	Depleted	3.8 ± 0.2	3.8 ± 0.1	4.2 ± 0.2^‡‡‡^	3.7 ± 0.2
Na^+^ (mmol·l^–1^)^§§§^	Loaded	140.3 ± 2.1	140.3 ± 1.4	141.4 ± 1.9^#^	140.7 ± 1.6
	Depleted	140.4 ± 1.7	140.4 ± 1.9	141.7 ± 1.4^##^	140.6 ± 1.5
pH^§§§^	Loaded	7.37 ± 0.02	7.36 ± 0.01	7.33 ± 0.04	7.33 ± 0.01^#^
	Depleted	7.37 ± 0.01	7.37 ± 0.02	7.35 ± 0.04	7.34 ± 0.02^##^
Glucose (mmol·l^–1^)	Loaded	4.8 ± 0.3	4.8 ± 0.4	4.9 ± 0.3	5.0 ± 0.4
	Depleted	4.8 ± 0.3	4.8 ± 0.4	4.8 ± 0.3	4.9 ± 0.4

Arm-venous plasma pH and plasma concentrations of K^+^, Na^+^, lactate and glucose were determined in the trials with the glycogen-loaded and glycogen-depleted legs at rest (pre exercise), during and post intense exercise to task failure. Data are expressed as means ± SD. N = 7 for all measures. ^§§§^Indicates a main effect of time (*P* < 0.001). ^†^Indicates a main effect of treatment (*P* < 0.05). ^‡‡‡^(*P* < 0.001) and ^‡^(*P* < 0.05) significantly different from all other time points. *(*P* < 0.05) significantly different from the loaded leg. ^#^(*P* < 0.05), ^##^(*P* < 0.01), ^###^(*P* < 0.001) significantly different from pre exercise.

## 4 Discussion

The main finding of the present study was that an exercise- and diet-induced low muscle glycogen content, compared to a high content, impaired performance during high-intensity one-legged knee extensor exercise, with an approximately 40% reduction in the time to task failure. Furthermore, resting muscle contractile characteristics, determined by the MVC as well as the electrically-induced single-twitch maximal force, RFD, and rate of relaxation, were unaffected by the glycogen content. Despite the higher muscle glycogen content in the glycogen-loaded condition, the single-twitch maximal force, RFD, and rate of relaxation were impaired to a greater extent at task failure than in the glycogen-depleted condition, whereas the MVC was similar. This suggests that a longer exercise duration in the glycogen-loaded leg was a decisive factor for impaired electrically induced contractile characteristics, while the MVC was unaffected by differences in the exercise duration. Manipulation of the muscle glycogen content did not affect the NKA content, whereas the exercise-induced increase in nonspecific phosphorylation of the NKA activity regulating FXYD1 protein after 5–6 min of cycling tended to be augmented in the glycogen-loaded leg.

### 4.1 Low glycogen content impairs continuous brief intense exercise performance

In this study, we showed, for the first time, that exercise-induced low muscle glycogen content impairs performance during continuous brief intense exercise lasting approximately 5 min (2.6 vs. 4.3 min). This supports the findings of Balsom et al. (1999) that the capacity to perform 15 repeated 6 s intense cycle intervals is impaired with low (180 mmol·kg_d.w._^−1^) compared to normal (397 mmol·kg_d.w._^−1^) glycogen levels (Balsom et al., 1999), albeit with markedly different exercise protocols. In contrast, elevated glycogen content compared to normal (approximately 700 vs. 350 mmol·kg_d.w._^−1^) does not affect the time to exhaustion during intense exercise lasting approximately 3 min (Bangsbo et al., 1992), indicating that low glycogen content has a detrimental effect, whereas high glycogen content does not improve performance during continuous intense exercise. The importance of the muscle glycogen content in relation to endurance exercise performance is well established (Allen et al., 2008) and is exemplified by a strong correlation between the cycling capacity (75% of $\dot{V}$ O_2max_) and initial muscle glycogen content (Bergstrom et al., 1967) and by improved endurance exercise performance (>90 min) with elevated muscle glycogen levels (Hawley et al., 1997). Thus, our finding of an exercise performance impairment during short-duration intense exercise with glycogen depletion aligns with that of prolonged exercise, although the present study did not compare with performance during normal resting glycogen content. Taken together, these findings are consistent with the suggestion that a critical level of muscle glycogen content exists, below which muscle function is compromised (Ortenblad et al., 2011), irrespective of the exercise type.

### 4.2 Glycogen content and muscle contractile characteristics

Reduced glycogen content may impair muscle function at major ATP-dependent sites, including myosin ATPases, SR Ca^2+^ ATPases, and NKAs (Nielsen et al., 2022). However, from a mechanistic perspective, how low glycogen content impairs intense exercise capacity remains unclear. A glycogen content of less than approximately 300 mmol·kg_d.w._^−1^ can impair *in vitro* Ca^2+^ release rates in SR vesicles (Ortenblad et al., 2011) and reduce the tetanic cytosolic Ca^2+^ concentration and force output (Chin and Allen, 1997). Our findings that the MVC and electrically induced twitches in a non-fatigued human major muscle group are unaffected by severely depressed muscle glycogen content suggest that sufficient Ca^2+^ is released for maximal activation of the contractile components, indicating that *in vivo* rates of SR Ca^2+^ release and reuptake are not impaired, at least in non-fatigued muscles.

At task failure from the intense one-legged knee extensor exercise, the MVC was similar in the two trials and tended to be higher in the glycogen-loaded leg (Table 1) despite the longer exercise duration. Whether this was caused by a faster gradual reduction in MVC in the glycogen-depleted trial or a similarly fast initial MVC reduction in both trials remains unclear. However, we recently reported that the loss of twitch force (Rannou et al., 2019), RFD, and rate of relaxation (Rannou et al., 2021) during a similar exercise protocol occurs gradually, with a fast initial component followed by a slower component. Based on these observations, it is expected that the markedly shorter exercise time in the glycogen-depleted leg is associated with a less pronounced loss of evoked twitch contractile properties (Table 2). Thus, a similar MVC observed at task failure in the present study may be caused by a faster decay of muscle contractile ability in the glycogen-depleted trial, even though the electrically evoked twitch muscle contractile characteristics seem to be dependent on the exercise duration. Noteworthy, the glycogen concentrations were not determined concomitant with the contractile characteristics at task failure from the one-legged exercise, however since subjects did not receive carbohydrates during the experiment, we expected similar differences in glycogen content between the two legs, as observed after the two-legged cycling.

The recovery patterns of MVC, single-twitch maximal force, RFD, and rate of relaxation were similar in the glycogen-loaded and glycogen-depleted legs. However, a significant treatment effect was apparent and appeared to be related to the less compromised muscle function, as determined by electrical stimulations (Table 2) in the glycogen-depleted trial at 60 s of recovery and concomitant similar MVC with a tendency to be higher in the glycogen-loaded leg. The reason for this is unclear but may be related to an expected higher severity of metabolic disturbances with a longer exercise duration (Juel, 1997; Sugaya et al., 2011; Hostrup et al., 2021), as indicated by a slightly lower muscle homogenate pH in the glycogen-loaded trial (Figure 3B). Taken together, these results suggest that the recovery pattern from task failure is unrelated to the prevailing glycogen content.

### 4.3 Protein abundance and phosphorylation

Manipulating the glycogen content did not alter either the NKAα_1_, α_2_, and β_1_ isoform or FXYD1 protein abundances or the total NKA content determined by [^3^H]-ouabain binding. Thus, changes in abundance *per se* of the most important K^+^-transporting protein (Hostrup et al., 2021) did not play a role in the glycogen-induced difference in muscle performance. Importantly, nonspecific FXYD1 phosphorylation was lower at rest in the glycogen-loaded leg than in the glycogen-depleted leg (Figure 5A). Furthermore, from rest to the end of the two-legged cycling, the nonspecific FXYD1 phosphorylation increased in both legs, as seen previously (Benziane et al., 2011; Thomassen et al., 2011; Thomassen et al., 2013; Thomassen et al., 2016), and this increase tended (*P* = 0.06) to be augmented in the glycogen-loaded compared to in the glycogen-depleted leg, as indicated by a greater decrease in signal intensity (−0.33 ± 0.07 vs. −0.19 ± 0.07, respectively). In contrast, FXYD1ser^68^ phosphorylation did not increase (Figure 5B), most likely due to concomitant higher FXYD1ser^63^ and thr^69^ phosphorylation (Figures 5C,D), which influenced the binding of the AB_FXYD1ser68 antibody, blunting and underestimating the FXYD1ser^68^ phosphorylation measurements (Fuller et al., 2009; Thomassen et al., 2016). Thus, augmented FXYD1 phosphorylation may indicate a greater increase in NKA activity (Bibert et al., 2008) during intense exercise in the glycogen-loaded leg, which may have improved K^+^ and Na^+^ handling (Gunnarsson et al., 2013; Thomassen et al., 2016) and attenuated the increase in the rate of arm venous [K^+^]. However, further investigation is warranted, including NKA activity determination. AMPK activity, determined by ACCβser^211^ phosphorylation (Birk and Wojtaszewski, 2006), was increased by exercise but was unaffected by the glycogen levels (Figures 6A,B) and therefore probably did not differentially affect the NKA activity in the glycogen-loaded and -depleted legs.

The abundance of NHE1 was higher in the depleted leg than in the loaded leg, which may have counteracted the findings of impaired K^+^ handling. However, a higher abundance of NHE1 is not necessary for better K^+^ handling and improved performance (Bangsbo et al., 2009; Gunnarsson et al., 2013). Thus, changes in NHE1 abundance did probably not affect the overall effects of glycogen depletion in this study.

### 4.4 Exercise-induced metabolic disturbances and potential interaction between observations

The small changes in plasma [K^+^] and [Na^+^] after intense two-legged cycling and one-legged knee extensor exercise (Table 3) most likely reflect the large gradient between the antecubital venous sampling site and the active muscles as well as the post-exercise sampling time (McKenna et al., 2024). One-legged knee-extensor exercise to exhaustion with a similar duration showed an interstitial [K^+^] of 8.8–13.7 mM (Nordsborg et al., 2003), while the arm-venous plasma [K^+^] reached the same concentration in both trials (Table 3) with a shorter exercise duration in the glycogen-depleted trial. A potential consequence of increased interstitial [K^+^] is reduced muscle cell excitability (Cairns et al., 1995), which has been related to the development of fatigue, especially in type II fibers (Nielsen et al., 2017), due to the intermittent firing of action potentials during trains or completely inexcitable fibers (Lindinger and Cairns, 2021). Thus, far greater increases in interstitial [K^+^] are expected than the observed increases in venous [K^+^], and they may reach levels close to where a depressive effect may be seen, especially since intracellular metabolic stress would be expected (Renaud et al., 2023).

The *in vivo* MVC is assessed over a longer duration (3–4 s) than electrically induced isometric twitches (2–300 ms), involves voluntary tetanic activation, is conducted as trains, and is often initiated by a high-frequency doublet (i.e., two close spikes <5 ms apart) discharge (Van Cutsem et al., 1998). Therefore, the MVC during contractions would generally be more sensitive to disturbances in the ability to regulate excitability than isolated peripheral muscle function. During exercise, the same decrease in MVC was obtained over a longer duration in the glycogen-loaded leg, and in recovery, the MVC in the loaded leg tended (*P* = 0.06) to be higher, especially immediately after task failure (Table 1). This occurred concomitantly with greater impairments in peripheral contractile muscle function, probably due to more severe metabolic intracellular disturbances (e.g., lower pH; Figure 3B) and higher P_i_ and lactate, in part given the approximately 65% longer exercise duration at similar exercise intensities (Juel, 1997; Sugaya et al., 2011; Hostrup et al., 2021), which impaired Ca^2+^ handling and muscle twitch function (Cheng et al., 2018). The attenuated decrease in MVC in the loaded leg may have resulted from improved protection of excitability during the 3–4 s of isometric contractions, partly due to better ion handling in the recruited fibers. Notably, shortly after task failure from intense exercise, the K^+^ balance and membrane potential may be partially restored, as seen in both humans (Vollestad et al., 1994) and mice, where the resting membrane potential recovers with a time constant of 0.9 and 1.5 min after repetitive stimulation in the soleus and EDL muscles, respectively (Juel, 1986). This might explain why differences in the MVC between the glycogen-loaded and glycogen-depleted legs in this study were most pronounced immediately after exercise (Table 1) but probably also occurred during exercise.

Directly comparing stimulation motor output between humans and rodents is difficult because of size differences and because rodents have approximately 3–4 times higher stimulation frequencies than humans (Manuel et al., 2019). In mouse fast-twitch muscles, initial stimulation frequencies occur above 250 Hz (Gorassini et al., 2000). While in rats during train stimulations, fiber membranes become partially depolarized (Fraser et al., 2011), which increases the repriming period in fast-twitch muscles from 4.1 to 7.8 ms (Dutka and Lamb, 2007). In addition, glycogen depletion can increase the action potential repriming period from 4.5 to 5.3 ms (Watanabe and Wada, 2019) and from 4 (25th percentile:75th percentile, 4:5) to 8 (7:10) ms in rat EDL skinned fibers (Jensen et al., 2020). Thus, the combined effect of exercise-induced membrane depolarization and glycogen depletion may increase the repriming period sufficiently to influence the force outcome induced by stimulation frequencies above approximately 100 Hz (corresponding to 10 ms intervals). This is most crucial in type II fibers with faster glycogen depletion at high intensities (Gollnick et al., 1974) and less protected excitability (Pedersen et al., 2009), despite more pronounced FXYD1 phosphorylation (Thomassen et al., 2013). The electrically induced single twitch force and RFD determined post-exercise are unaffected because of the short time frame of 2–300 ms. In contrast, during the MVC (3–4 s), an increase in the action potential repriming period affects the force production, which is more pronounced during and immediately after intense exercise than during resting conditions. Due to the longer single-legged exercise duration compared to that of the glycogen-depleted leg, the augmented increase (*P* = 0.06) in nonspecific FXYD1 phosphorylation in the glycogen-loaded leg may induce an even greater nonspecific FXYD1 phosphorylation, which increases over time during continuous exercise (Thomassen et al., 2011) and thus suggests a possible greater NKA activity during exercise in the loaded leg. In support of this, differences in performance, determined using the pedaling frequencies, were only significant in the last part of the 6 s intense exercise sessions in glycogen-depleted participants (Balsom et al., 1999). Furthermore, a pronounced role of glycogen in excitability is observed *in vitro* in rat skeletal muscle when SR Ca^2+^ release is mediated by action potentials (Nielsen et al., 2009), while no effect is observed by direct stimulation of the voltage sensors by Na^+^ depolarization (Goodman et al., 2005), indicating that the coupling between glycogen and fatigue is at least partly due to t-tubular system depolarization. In mouse muscles, a synergistic interaction between increased extracellular [K^+^] and decreased muscle glycogen content has been demonstrated (Cairns and Renaud, 2023). Interestingly, a high abundance of NKA, especially the NKAα_2_ isoform, as well as the SR Ca^2+^ release channels are co-localized within the diffusion-restricted space between the SR and t-tubular system, and in rat skeletal muscles, the NKAs utilize glycogen only from the intramyofibrillar compartment (Nielsen et al., 2022). Furthermore, in humans, high-intensity exercise reveals a compartmentalized glycogen metabolism, including a higher glycogen breakdown rate in type II fibers than in type I fibers, especially in the intra-and intermyofibrillar glycogen fractions (Vigh-Larsen et al., 2022). Together, this indicates that preserved cell membrane excitability, possibly due to higher NKA activity suggested by increased FXYD1 phosphorylation, may play an important role in the improved high-intensity exercise performance in the glycogen-loaded leg; however, this warrants further exploration.

In conclusion, we showed that exercise- and diet-induced low muscle glycogen content impaired continuous high-intensity exercise performance lasting a few minutes. Resting muscle contractile characteristics were unaffected by the muscle glycogen content, whereas at task failure, the electrically-induced single-twitch maximal force, RFD, and rate of relaxation were impaired in the glycogen-loaded leg, probably because of the longer exercise duration. Concomitantly, the MVC was similar and tended to be augmented in the glycogen-loaded leg. Phosphorylation of the NKA activity-regulating FXYD1 tended to be more pronounced in the glycogen-loaded leg during exercise. However, how low glycogen content impairs brief and intense exercise capacity remains unclear.

### 4.5 Limitations

In this study, muscle glycogen status was manipulated using a combination of dietary control and exercise timing. We observed clear differences in the muscle glycogen content in the legs. However, the glycogen-depleted leg was exercised the evening before the experiment, which may have induced inflammation and increased production of reactive oxygen species, potentially affecting the measurements the following day. Regardless, the influence of exercise timing was not observed at rest on the experimental day, as determined by both the MVC and electrical stimulations. In addition, both legs completed the two-legged intense cycling task before the one-legged knee extensor exercise to task failure, which was used to determine performance.

Furthermore, because of difficulties in obtaining femoral blood samples during cycling, antecubital venous sampling was used. Although this sampling site was non-optimal when investigating leg exercises, any detected differences would have been relevant. As well, due to the many different measurements obtained, sample size and statistical power may be limited for some data, especially for muscle pH and plasma metabolites. Thus, some constraints may be taken in interpretation of blood values, muscle content and muscle contractile characteristics.

Finally, the absolute muscle pH values reported in this study were higher than expected. However, the pH was determined using homogenates at room temperature, which probably induced higher values than intracellular pH at 37°C due to evolution of CO_2_ in homogenates (Spriet et al., 1986). As well, previous studies have reported individual resting pH values near 7.4 (Mannion et al., 1993; Mannion et al., 1995). Although the reported muscle homogenate pH values were high, the patterns between the legs and from rest to task failure are still valuable.

## Data Availability

The raw data supporting the conclusions of this article will be made available by the authors, without undue reservation.
